# Study protocol: implementation of a computer-assisted intervention for autism in schools: a hybrid type II cluster randomized effectiveness-implementation trial

**DOI:** 10.1186/s13012-016-0513-4

**Published:** 2016-11-25

**Authors:** Melanie Pellecchia, Rinad S. Beidas, Steven C. Marcus, Jessica Fishman, John R. Kimberly, Carolyn C. Cannuscio, Erica M. Reisinger, Keiran Rump, David S. Mandell

**Affiliations:** 1 Center for Mental Health Policy and Services Research, Perelman School of Medicine, University of Pennsylvania, 3535 Market St., 3rd Floor, Philadelphia, PA 19104 USA; 2 The Wharton School, University of Pennsylvania, 2109 Steinberg-Dietrich Hall, 3620 Locust Walk, Philadelphia, PA 19104 USA; 3 Section on Public Health, Perelman School of Medicine, University of Pennsylvania, Anatomy and Chemistry Room 145, 3620 Hamilton Walk, Philadelphia, PA 19104 USA

**Keywords:** Effectiveness-implementation trial, Exnovation, De-implementation, Computer-assisted intervention

## Abstract

**Background:**

The number of children diagnosed with autism has rapidly outpaced the capacities of many public school systems to serve them, especially under-resourced, urban school districts. The intensive nature of evidence-based autism interventions, which rely heavily on one-to-one delivery, has caused schools to turn to computer-assisted interventions (CAI). There is little evidence regarding the feasibility, effectiveness, and implementation of CAI in public schools. While CAI has the potential to increase instructional time for students with autism, it may also result in unintended consequences such as reduction in the amount of interpersonal (as opposed to computerized) instruction students receive. The purpose of this study is to test the effectiveness of one such CAI—TeachTown—its implementation, and its effects on teachers’ use of other evidence-based practices.

**Methods:**

This study protocol describes a type II hybrid cluster randomized effectiveness-implementation trial. We will train and coach 70 teachers in autism support classrooms in one large school district in the use of evidence-based practices for students with autism. Half of the teachers then will be randomly selected to receive training and access to TeachTown: Basics, a CAI for students with autism, for the students in their classrooms. The study examines: (1) the effectiveness of TeachTown for students with autism; (2) the extent to which teachers implement TeachTown the way it was designed (i.e., fidelity); and (3) whether its uptake increases or reduces the use of other evidence-based practices.

**Discussion:**

This study will examine the implementation of new technology for children with ASD in public schools and will be the first to measure the effectiveness of CAI. As importantly, the study will investigate whether adding a new technology on top of existing practices increases or decreases their use. This study presents a unique method to studying both the implementation and exnovation of evidence-based practices for children with autism in school settings.

**Trial registration:**

NCT02695693. Retrospectively registered on July 8, 2016.

## Background

Autism spectrum disorder (ASD) is characterized by impairments in socialization and communication and is accompanied by highly restricted interests and repetitive behaviors [1]. The number of children with autism in special education has increased an average of 17% a year for the past 15 years [2]. This increase places a significant financial burden on schools. Evidence-based autism interventions are expensive, requiring skilled interventionists working with children individually or in small groups for up to 25 h per week [3]. The average annual expenditure per pupil with autism is three times that of children in general education and twice that of other children in special education [5]. While there is little description of services associated with this elevated expenditure [4, 6–9], it is likely that much of the increase comes from the use of these intensive interventions [10].

In recent years, computer-assisted interventions (CAI) have gained popularity as a method for educating children with ASD [11]. Many CAI have integrated evidence-based instructional strategies for children with ASD [7, 12–17]. CAI is appealing to under-resourced schools because of the potential to provide cost-effective individualized instruction while freeing up teachers to provide concurrent group instruction.

Most studies of CAI are descriptive and exploratory, and employ small samples with single-subject research designs [16, 18]. Preliminary findings indicate promising results for academic, social, and language outcomes, with anecdotal improvements in behavior and compliance during instructional time [19–21]. Overall, however, the use of CAI for students with ASD has outpaced the evidence for its efficacy [12]. A notable exception is TeachTown: Basics, a CAI that includes computerized lessons combined with teacher-delivered interpersonal instructional activities. In a randomized trial, students who received instruction using TeachTown improved more on language and cognitive outcomes than students in a control group after 3 months of intervention [22]. The study sample was small, however, and the study was conducted in only a few classrooms.

New technologies like TeachTown could have unintended consequences or be implemented in unexpected ways. For example, teachers may choose to implement some components of TeachTown and not others. They also may perceive that TeachTown can substitute for existing practices and change other instructional approaches as a result. Understanding the factors that affect the implementation of CAI and how the implementation of CAI affects teachers’ use of other evidence-based practices is critical for evaluating their effectiveness, feasibility, and sustainability in under-resourced public schools.

The purpose of this hybrid effectiveness-implementation [23] study is to (1) assess the effectiveness of the TeachTown program in improving the outcomes of youth with ASD using a randomized trial in an urban public school district, (2) understand how teachers implement this new technology within their classrooms and the factors that affect implementation, and (3) evaluate how existing evidence-based practices (EBP) change when a new technology is introduced.

**Fig. 1:**
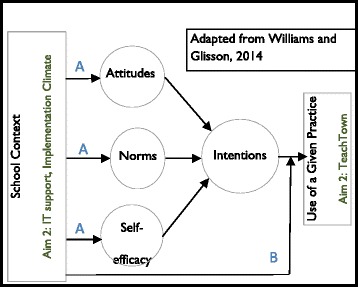
Conceptual model

We hypothesize that TeachTown will be associated with improvements in children’s cognitive ability and academic skills. Our hypotheses about factors affecting TeachTown implementation, and what will happen to existing practices when TeachTown is introduced, are driven by a conceptual model that combines well-established theories of behavior with organizational variables. Williams and Glisson propose a similar model that integrates organizational context and behavioral intention [24], which we have expanded by adding the determinants of intention: attitudes, norms, and self-efficacy (Fig. 1).

At the core of our model is the theory of planned behavior, which posits that individuals’ intention to perform a certain behavior is the most proximal determinant of that behavior, when individuals have the ability to act on their intentions. The determinants of intention are attitudes (e.g., whether one “likes” or “dislikes” using a given EBP), norms (e.g., whether one perceives that using a given EBP is viewed as appropriate by others), and self-efficacy (e.g., whether one believes that one has the necessary skills to perform the EBP). This model is commonly used to predict health behaviors and is gaining currency as a tool for understanding mental health clinicians’ adoption of evidence-based practices [25], as well as teachers’ behavior, including use of educational technology [26] and different curricular and teaching approaches [27–31].

This model motivates two sets of hypotheses. First, we hypothesize that a strong organizational context, defined as climate for innovation implementation [32] will positively influence the determinants of intention and result in greater use of TeachTown, as will the presence of greater IT support (pathway A). Second, we hypothesize that schools’ organizational context and IT support will moderate the association between intentions and behavior, such that, among teachers with strong intentions to use TeachTown, better support will result in greater use (pathway B).

For the past 8 years, our team has provided extensive training and coaching to autism support teachers in the use of several EBPs for children with ASD. The second set of hypotheses relate to teachers’ use of the EBPs in which we have trained them (aim 3). For this aim, we conceptualize TeachTown as a new technology that affects the organizational context and teacher behavior. TeachTown may influence teachers’ attitudes, norms, and self-efficacy about the use of existing EBP (pathway A) and moderate the association between their intentions to use these practices and their behavior (pathway B). Specifically, we hypothesize that introducing TeachTown will negatively affect teachers’ attitudes, norms, and perceptions about using existing EBP, and that teachers will “exnovate” existing practices when they adopt TeachTown. Exnovation refers to the “process whereby an organization decides to divest itself of an innovation that it had previously adopted.” [33–35] Introducing a new technology may result in one of three exnovation outcomes: full exnovation (the new technology completely replaces the previous one so that there is no trace of the previous practice), partial exnovation (the new technology replaces parts or components of the previous practice), or no exnovation (the new technology does not replace the previous practice). We hypothesize that the introduction of TeachTown will result in partial exnovation of EBPs. Teachers may reduce their use of EBP because they think that TeachTown is a reasonable (and easier) substitute for practices like one-to-one instruction or data collection. Our second competing hypothesis is that introducing TeachTown changes the organizational context by giving teachers a tool to provide unsupervised instruction to some students, which would increase the time the teacher has to work with other students. In this case, we would expect use of EBP to increase in classrooms with TeachTown.

TeachTown may be a feasible intervention that improves outcomes for children with ASD in public schools that is well suited for under-resourced settings. On the other hand, there may be negative consequences of implementing CAI, such as reduced use of existing EBPs, which should be weighed against potential benefit. The proposed study offers a novel opportunity to test the effectiveness and implementation of a promising CAI and concurrently increase our understanding of what happens to existing practices when new ones are introduced. Accordingly, we rely on a hybrid effectiveness-implementation design [36], in which the primary outcome is effectiveness, but where we also examine the association of organizational and teacher characteristics with TeachTown and EBP implementation.

## Methods

### Setting

The study will occur in the School District of Philadelphia, the eighth largest school district in the country. The majority of students served by the school district are ethnic minorities (69%); 75% live below the poverty line. The district has attempted to improve access to evidence-based interventions for students with ASD by supporting the implementation of a comprehensive treatment package (Strategies for Teaching based on Autism Research: STAR), consisting of a set of evidence-based practices for the treatment of ASD, in autism support classrooms. As part of the current study, we will provide consultation and training to teachers in the use of the EBP in which we have provided training for the past 8 years. Our coaches provide didactic training in a group setting, as well as in vivo coaching in each classroom regarding the use of the EBPs.

### Participants

#### Teachers

Teachers in the School District of Philadelphia’s K-2 autism support classrooms (*n* = 83) will be recruited. Teachers will participate in TeachTown training as part of their professional development but will not be required to participate in this study. We anticipate recruiting 80% of teachers (69 classrooms). Teachers will be randomly assigned to one of two conditions during year 1 of the study: TeachTown and waitlist. Teachers in the waitlist condition will receive training and support in the use of TeachTown in year 2 of the study.

#### Children and their families

By district regulation, children in K-2 autism support classrooms will be between the ages of 5 and 8. The recruitment goal for the study is 4–5 students in each of the 69 classrooms (which usually have 8 students), for a total of 276 participants. Inclusion criteria are that children have an educational classification of autism and be enrolled at least half time in a K-2 autism support classroom. The one exclusion criterion is if the primary caregiver does not speak English or Spanish, which would exclude <1% of eligible participants.

### Overview of intervention

TeachTown: Basics is a CAI that includes off-computer interpersonal activities, automatic data collection and reporting, and a note system for communication with the child’s team. The curriculum is designed for children developmentally aged 2–7 years, which describes the vast majority of our potential sample.

#### *Computer-assisted instruction*

The computer lessons incorporate the principles of ABA, using a discrete trial format [37], in which the student is provided with a specific instruction, and selects the correct response. Correct responses are immediately reinforced using animated reward games, verbal praise, and graphics. The lessons use specific prompting procedures, such as fading and highlighting the correct answers, to promote success. The curriculum progresses through five levels, and students’ progress at their own pace. The curriculum content addresses six domains: (1) adaptive skills, (2) cognitive skills, (3) language arts, (4) language development, (5) mathematics, and (6) social emotional skills. Progress monitoring is part of the program. Students complete pre-tests and must demonstrate mastery before progressing to the next lesson. Teachers are asked to have the child spend 20 min per day on the software either with the teacher, an aide, or independently.

#### *Off-line activities: interpersonal lessons*

Teachers provide interpersonal lessons via direct instruction. These lessons focus on the same areas targeted in the CAI activities; however, the lessons are designed to promote expressive language and interaction skills. Lesson plans and cues for instructional delivery are included with the program.

### Teacher training in TeachTown

Consistent with best practices [38–40], teachers in the intervention group (and the control group in year 2) will receive 2 days of didactic training at the start of the school year, with experiential components, webinars throughout the school year, and monthly consultation. Training and monthly consultation in TeachTown will be provided by the program developers.

#### Aim 1: effectiveness: How does TeachTown affect student outcomes?

We will examine to what extent TeachTown is associated with student gains in cognitive ability, language skills, school readiness, and social skills. We hypothesize that use of TeachTown will be associated with improved student outcomes in these domains.

### Measures

Our primary outcome of interest, in keeping with recent randomized trials of autism interventions, is cognitive gain. Other selected measures are tied to the domains in which TeachTown purports to affect change (see Table 1).

**Table 1:** List of measures, respondents, and time points for data collection

Measure	Respondent	Time point
Aim 1: Effectiveness		
Differential Ability Scales, 2nd Edition (DAS-II)	Direct observation of student functioning completed by graduate student or clinician	Beginning and end of year 1
Bracken Basic Concept Scales, 3rd Edition	Direct observation of student functioning completed by graduate student or clinician	Beginning and end of year 1
Autism Diagnostic Observation Schedule—2nd edition (ADOS-2)	Direct observation of student functioning completed by graduate student or clinician	Throughout year 1
Adaptive Behavior Assessment System, 2nd Edition (ABAS-II)	Teacher rating form of child functioning	Beginning and end of year 1
Pervasive Developmental Disorder Behavior Inventory (PDD-BI)	Teacher rating form of child functioning	Beginning and end of year 1
Social Communication Questionnaire (SCQ)	Parent rating form of child functioning	Beginning of year 1
Family Demographic Questionnaire	Parent	Beginning of year 1
Teacher Demographic Questionnaire	Teacher	Beginning of year 1
Aim 2: Implementation		
TeachTown penetration	Computer software logs	Monthly
TeachTown fidelity	Direct observation of teacher behavior conducted by TeachTown staff	Monthly
Intentions and attitudes scale	Teacher rating scale	Mid-year years 1 and 2
Aim 3: Exnovation		
Existing EBP fidelity	Direct observation of teacher behavior conducted by trained research assistants	Monthly

*Cognitive ability* will be assessed using the Differential Ability Scales, 2nd Edition (DAS-II) [41]. The DAS-II assesses cognitive abilities that are important to learning and may be administered to children ages 2 years 6 months through 17 years 11 months across a broad range of developmental levels. A member of the assessment team will administer the DAS-II to students in the study at the beginning and end of the academic year in year 1.

*Adaptive skills* will be measured using the Adaptive Behavior Assessment System, 2nd Edition (ABAS-II) [42]. The ABAS-II uses a behavior-rating format to assess adaptive behavior and related skills for individuals, birth through 89 years of age. Teachers will complete the ABAS-II at the beginning and end of the academic year in year 1.

*School readiness skills*, including *language arts*, *language development*, and *mathematics*, will be measured using the Bracken Basic Concept Scale—3rd Edition [43]. This scale assesses knowledge of concepts associated with pre-academic skills, such as colors, letters, numbers/counting, sizes, comparisons, and social concepts like social relationships and emotions [44–46]. A member of the assessment team will administer the Bracken at the beginning and end of the school year in year 1.

*Social skills and pragmatic language* will be measured using the Pervasive Developmental Disorder Behavior Inventory (PDD-BI) [47]. The PDD-BI is a rating scale that assesses problem behaviors, social skills, language skills, and learning or memory skills in children who have been diagnosed with autism. It can be used with children between the ages of 1.6 and 12.5 years. The teacher report version of the PDD-BI will be administered by a member of the assessment team at the beginning and end of the academic year in year 1.

#### *Autism classification*

All children in the study will have an educational classification of ASD provided by a school psychologist as part of their educational evaluation. Autism diagnoses will be confirmed in our sample using the Social Communication Questionnaire (SCQ) [48], a parent-report measure that examines the presence of autism spectrum disorder (ASD) symptoms in children, and the Autism Diagnostic Observation Schedule—2nd edition (ADOS-2), administered to 20% of the sample.

#### *Demographic survey*

A parent or guardian will complete a form that includes demographics, family composition, education, and financial resources using the relevant US Census Survey questions.

### Data collection

#### *Direct observation measures*

Members of the assessment team will visit each school at baseline and the end of the academic year to administer the DAS-II and Bracken. The team will meet weekly to maintain 90% reliability on these measures.

#### *Parent report measures*

Parents will complete the demographic survey and SCQ at baseline. Parents will be requested to mail in the form, with follow-up phone calls to increase the response rate. They will receive $50 for completing the forms.

#### *Teacher report measures*

Classroom teachers will complete the ABAS and the PDD-BI for each student enrolled in the study at the beginning of the year and again at the end of the academic year in year 1. Teachers will receive $20 per child for each wave of data collection.

### Data analyses

Analysis will be based on randomization of classrooms to TeachTown (*n* = 35) or control (*n* = 34). Outcomes will be analyzed at the student level across time. Outcome differences will be estimated with longitudinal nested linear models with random effects for classroom and student [49–51]. All outcome measures are continuous variables.

We also will examine several potential moderators of TeachTown impact, including: (1) symptoms at baseline (measured by the PDDBI); (2) cognitive functioning at baseline (measured by the DAS); (3) penetration (whether the consented student received TeachTown instruction based on the electronic logs); (4) fidelity of program implementation (direct observation of generalization lessons, described in aim 2); (5) use of the EBPs described in Aims 3; and (6) family characteristics, including family composition and parent education and income. Potential moderators will be included in the model as main effects and as interaction terms with time and treatment group factors in the model. The three-way interaction between time, treatment group, and the moderator will be used to assess the presence and magnitude of the moderating effect. Because both TeachTown and EBP fidelity may vary over the course of the year for each student, we will use growth mixture models to categorize fidelity trajectories at the student level.

#### Aim 2: implementation: How do teachers use TeachTown, and what factors are associated with its use?

We will measure implementation of the TeachTown program using a subset of the implementation outcomes described by Proctor and colleagues [53]. Specifically, we will measure teachers’ fidelity to the program manual and penetration of use across classrooms. We hypothesize that the percentage of students using TeachTown and fidelity will vary among teachers and over time. Both will be influenced by teachers’ intentions to use TeachTown and determinants of those intentions (attitudes, norms, and self-efficacy). Because this aim focuses on teacher behavior (rather than student outcome), we aggregate penetration and fidelity to the teacher level.

### Measures

#### *TeachTown penetration*

Teachers’ use of TeachTown will be measured using the logs the TeachTown software produces.

#### *TeachTown Fidelity*

Teachers’ accurate use of TeachTown will be measured monthly via direct observation by coaches from the TeachTown program using a fidelity checklist created by the program developers.

### Independent variables

#### *Measures of intention and determinants of intention*

A questionnaire will use validated, standardized item stems to measure intentions, attitudes, norms, and self-efficacy regarding use of TeachTown online and offline (separately). The stems for each question were designed to be adapted for study of any behavior and have been used to successfully predict a large variety of behaviors [54–59].

*Teachers’ intentions* to use TeachTown online and offline components for instruction of students with ASD will be measured by two items (e.g., “How likely is it that you will use TeachTown for online instruction of students with ASD?”). Scaled response options will range from 1 (very unlikely) to 7 (very likely). If highly correlated, the two measures of intention will be aggregated for each behavior.

*Teachers’ attitudes,* or the extent to which one “likes” or “dislikes” using TeachTown components, will be measured by six (7-point) bipolar adjective scales, scored −3 to +3. For example, scales will allow respondents to rate using a TeachTown component as extremely unpleasant–extremely pleasant and as extremely wise–extremely foolish. The mean score across the six items will constitute our measure of attitudes toward using a TeachTown component.

*Teachers’ perceived norms* will be measured using two standard measures that capture perceptions of normative pressure. For example, respondents will be asked to rate on a 7-point scale the perception that most autism support teachers will use TeachTown online. If highly correlated, the two measures will be aggregated.

*Teachers’ self efficacy* will be measured by asking respondents to rate, on a 7-point scale, the statement, “If I really wanted to, I could use TeachTown online in my classroom” as likely/unlikely.

*Other covariates of interest* include years of teaching experience (measured through self-report) and staff-to-child ratios in the classroom (measured through direct observation).

### Data collection

The TeachTown company will provide anonymized monthly data on all students’ use of TeachTown. These data, aggregated to the classroom level, will allow us to measure penetration for all students in the classroom. Independent variables, including intentions and determinants of intention, will be measured immediately following the 2-day TeachTown training, so that teachers will have familiarity with the program before answering these questions.

### Data analysis

Analysis will take place using data from years 1 and 2, so that teachers in the control group are included to maximize statistical power. The unit of analysis is teacher behavior (*n* ≈ 69). The outcomes of interest are penetration and fidelity (i.e., trajectories and variability) within teacher. Penetration will be measured as a count of the number of students in each classroom that log in any time on TeachTown during the year. We will use Poisson regression to model this outcome, using the total number of students in the classroom as the log offset.

We will model variability in fidelity within teacher in how their students use TeachTown. This fidelity may vary over time. Therefore, we will also model trajectories of *variance* in TeachTown fidelity over time for each teacher, using multinomial regression with these same growth curve mixture models. This analysis will give us a measure of consistency of fidelity across students over time within each classroom.

We will use structural equation modeling to identify the relative contribution of attitudes, self-efficacy, and normative pressure about each EBP to explain variation in intention to use each EBP. This analysis will determine if a homogenous or heterogeneous set of factors influence intentions to use each EBP. For example, attitudes may primarily predict intentions to use one EBP but self-efficacy may be the best predictor for another EBP. This kind of information suggests causal pathways, and future lines of research can use this information to design interventions that work for specific (or multiple) types of EBPs [60, 61]. Summaries of model fit will include overall goodness-of-fit summaries (e.g., model chi-squared), as well as more focused indices of fit.

To test the extent to which organizational factors are associated with determinants of intention and the use of an EBP, we will build on the structural equation model described above. We will add organizational variables to the model and estimate pathways between organizational variables and attitudes, norms and self-efficacy, as pictured in Fig. 1. To test the moderating effect of organizational variables on the association between intention and use of an EBP, we will use ordinal logistic regression. EBP use will be treated as continuous, and moderation will be tested using interaction terms between intentions and organizational variables, with random effects for school to adjust for the non-independence of the data.

#### Aim 3: exnovation: How does use of existing EBPs change when TeachTown is introduced, and what factors influence this change?

We hypothesize that (1) teachers will decrease the amount of one-to-one teaching incorporated into their daily schedules; (2) teachers will collect less data regarding student performance; and (3) the amount of positive reinforcement, behavior correction, and visual schedules provided by teachers will remain the same. We also will test the hypothesis that TeachTown facilitates EBP use by providing unsupervised instruction for some students, and therefore moderating the association between intentions and use of TeachTown. We will examine whether current EBPs are facilitated or exnovated when TeachTown is introduced and use qualitative methods to understand stakeholder perspectives of exnovation, if relevant. In separate analyses, we will examine whether use of TeachTown affects intentions to use EBP and determinants of those intentions: attitudes, norms and self-efficacy.

### Overview of current EBPs

As part of our ongoing training and consultation efforts, we train and consult to teachers in five EBPs that are common across most classroom-based autism interventions: discrete trial training, pivotal response training, data collection, positive reinforcement for classroom management, and visual schedules.

*Discrete trial training* is implemented using an intensive one-to-one teaching session in a setting free from distractions. Discrete trial training generally involves the repeated practice of the same response for several successive teaching episodes, breaking down complex skills into component parts, and the use of reinforcers that are functionally unrelated to the response (e.g., providing a token for correctly identifying a car).

*Pivotal response training* uses one-to-one teaching but relies on a less structured teaching environment. It consists of loosely structured sessions that are initiated and paced by the child, take place in a variety of locations, and employ a variety of stimuli. For example, in a situation in which the student wants to play with a toy, he must explicitly request the toy from the teacher.

*Classroom data collection* is a critical component of autism intervention. Each teaching strategy has data sheets to track responses during one-to-one teaching and levels of prompts needed during daily routines.

*Positive behavior support for behavior management* comprises class-wide behavior management techniques. The goal of these strategies is to prevent challenging behavior before it occurs and maximize opportunities for learning. Positive behavior support strategies include the use of visual supports, clear and concrete instructions, timers and cues to signal transitions, and high rates of positive reinforcement for desired behavior paired with low rates of behavior correction for undesired behavior.

*Visual schedules* are used throughout the day. A daily visual schedule for each child is posted in prominent locations and reviewed daily. Visual schedules are used to increase independence and decrease frustration during transitions.

#### Quantitative approach

To examine changes in the use of EBP as a result of TeachTown, we will compare the use of EBP in the TeachTown group and control group in year 1. To address the questions of (1) whether increased use of TeachTown results in changes in the use of EBP, (2) TeachTown implementation changes intentions to use other EBP, and (3) whether TeachTown moderates the association between intentions and use of EBPs, we use data from year 1 for the intervention group and year 2 for the waitlist control, so measures will be from the first year that each classroom implements TeachTown.

### Quantitative measures

#### Dependent variables

Accuracy of EBP implementation will be assessed every other month through direct observation in each classroom using fidelity checklists which comprise the most commonly used fidelity measures for these intervention techniques. [52, 62–64] Bachelor’s level research assistants will be trained to 90% reliability on each fidelity measure through didactic instruction and coding of training videos prior to conducting any observations in the field.

*Delivery of one-to-one instruction: discrete trial training and pivotal response training* will be measured through teacher report and direct observation by trained RAs. How much each is used with each student will be measured through teacher report. We will measure accuracy of discrete trial training through monthly direct observations using a fidelity checklist. We will examine quantity and accuracy separately as dependent variables. We also will examine the product of “quantity × accuracy” consistent with our previous evaluations of fidelity for these EBPs [64].

*Data collection* will be measured through observation of completed data sheets. Trained research assistants will record the occurrence of data collection by recording the amount of data collected for each child since the last observation, based on the number of completed data sheets present.

*Positive behavior support strategies* will be measured through direct observation of teacher behavior during regular instructional routines by trained research assistants. RAs will measure teacher’s use of positive reinforcement for desired student behavior using a tool designed to record the amount of praise and behavior correction statements provided by teachers during a 10-min structured observation [65]. Reinforcement will be calculated as a ratio (i.e., the number of praise statements divided by the number of correction statements).

*Visual schedules* will be measured monthly by trained RAs through direct observations based on a fidelity checklist. Use of schedules will be calculated as a ratio of the teacher’s correct use of schedules for each student divided by the total number of students.

### Independent variables

*Measures of intentions and determinants of intentions* will be measured as described in aim 2, but asking about the EBPs of interest rather than TeachTown.

#### *Data collection*

Implementation climate and IT infrastructure will be measured at the beginning of each year. Measures of teachers’ intentions, attitudes, perceived norms, and self-efficacy regarding the use of the existing evidence-based practices will be measured in September and April of year 1 for the intervention group and year 2 for the control group. Surveys will be collected directly from the teacher.

#### *Quantitative data analysis*

The quantitative analysis will take place in three parts, each addressing one of the questions of interest. To address the question of whether TeachTown results in reduced use of EBP, we will compare the intervention and control groups in year 1. We will conduct the analysis at the teacher level. We will create three fidelity variables for each of the five EBPs, as we did in aim 2. The analyses will mirror those described in aim 2. To address the question of whether introducing TeachTown affects intentions to use EBPs, we will conduct similar analyses with intentions to use each of the EBPs at time 2 as the outcomes of interest. If intentions are determined to vary as a function of TeachTown use, we then will explore the effects of TeachTown on each of the determinants of intention.

### Qualitative approach: How do teachers view TeachTown and its effects on their use of EBP?

#### *Participants*

We will recruit 48 teachers. Our purposive sampling strategy is presented in Table 1 and ensures that we will reach saturation in each quadrant [66].

#### *Method*

Semi-structured interviews will be conducted with teachers at the end of years 1 and 2. These interviews provide textual data that can be analyzed for themes, patterns, and ultimately, grounded theory [67]. Standardized probes will be included in the interview guide so that consistency across interviews is maintained. Interviews will be conducted by RAs under the direction of experts in qualitative research. We also will examine if the decision to exnovate is a conscious process. Specifically, we will query teachers’ rationale for using TeachTown; how they view TeachTown in relation to the other EBPs (e.g., complement, replacement) and the utility and effectiveness of each. Interviews will be digitally recorded with the participants’ permission, professionally transcribed, and loaded into Nvivo 10.0 software for data management and analysis.

#### *Qualitative analysis*

Interviews will be analyzed using an integrated approach [45]. Transcripts will be analyzed in an iterative process based upon an integrated approach that incorporates both inductive and deductive features [68]. Through a close reading of eight transcripts, the investigators will develop a set of codes to apply to the data (i.e., inductive approach). A priori codes derived from the original research questions and previous literature will also be applied (i.e., deductive approach). A random subset of transcripts (20%) will be coded by two investigators, and inter-rater reliability will be expected to be at least 90% [69]. Each reviewer will produce memos including examples and commentary to reach consensus regarding newly derived, emergent themes that emerged from the codes [68]. Once the data are coded, codes will be summarized and examined for patterns to create a tentative theory about the data.

#### *Mixed methods analysis*

We will integrate the qualitative findings with quantitative measures of teacher fidelity and factors associated with fidelity. The design taxonomy is as follows: the structure is simultaneous (we will gather quantitative and qualitative data concurrently and weigh them equally: QUAN + QUAL); the function is complementarity (to elaborate upon the quantitative findings to understand the *process* of change teachers experience); and the process is connecting (having the qualitative data set build upon the quantitative data set) [70]. We will use mixed methods in two ways. First, we will use quantitative findings to identify patterns in the qualitative data. To do this, we will enter quantitative findings into Nvivo as attributes of each participant. Quantitative attributes will be used to categorize and compare important themes among subgroups. Then, as themes emerge from the interviews, we can use Nvivo to query whether the presence and quality of these themes differ among teachers with low, average, and high fidelity. Second, we can use the qualitative data to help interpret quantitative results, especially if there are counterintuitive findings.

#### *Statistical power for quantitative analyses*

The prior trial of TeachTown reports that students made gains in communication skills of a moderate effect [71]. Meta-analytic reviews of studies of other interventions for children with ASD report large intervention effects [4, 72–74]. Based on this literature, we conducted a power analysis to determine the effect sizes we could expect in our cluster randomized study of students nested within the 70 classrooms in the district. Using the Power and Precision software package [75], we calculate that for student outcomes we will have power of 80% to detect a moderate intervention effect of *d* = 0.43, assuming enrollment of four students per classroom, an intraclass correlation (ICC) of 0.2, and inclusion of one covariate in our regression model that explains 15% of the variation. For teacher outcomes, we will have 80% power to detect a larger effect of the intervention (*d* = .7) assuming a model with a single covariate (*r* ^2^ = .15). All models assume two tailed tests with alpha = 0.05.

Our secondary goal is to test the potential impact of moderating factors. With the proposed sample size, the study will have power of 80% to identify an effect size associated with moderators of *d* = 0.65 [71], a moderate-to-large effect. For teacher-level analyses, we are powered to test moderators with an effect size of *d* = 0.85, a large effect.

## Discussion

The present study contains several important innovations. First, it relies on a strong partnership between our research group and the School District of Philadelphia [76], a critical component of implementation research. Through this partnership, we have gained an in-depth understanding of the barriers and facilitators to EBP implementation, making this an ideal setting in which to examine the implementation of a new technology.

Second, despite the growing popularity of CAI, the proposed study represents the first large-scale effectiveness-implementation study of a specific CAI for children with autism. Third, our study is one of the first to combine psychological theories of behavior change with organizational theories to examine predictors of EBP implementation. This information will provide valuable insight into modifiable factors which may affect the implementation of new technologies and identify possible implementation targets.

Finally, our study would be the first prospective study to examine changes in the use of existing EBPs as new practices are implemented. As denoted by the call for more research on this topic [77], little is known about what happens to existing practices when new practices are introduced. We will systematically study how the process of exnovation [33] unfolds as a new technology is introduced by evaluating changes in teachers’ use of existing EBPs and understanding why, after the TeachTown program is introduced.

This study provides an important opportunity to systematically evaluate the effectiveness of a new intervention for students with autism, while providing valuable insights into strategies to support the implementation of existing practices in public schools.
